# A Pilot Study Investigating the Expression Levels of Pluripotency-Associated Genes in Rectal Swab Samples for Colorectal Polyp and Cancer Diagnosis and Prognosis

**DOI:** 10.1155/2021/4139528

**Published:** 2021-07-22

**Authors:** Ryan Wai-Yan Sin, Dominic Chi-Chung Foo, Deepak Narayanan Iyer, May Sau-Yee Fan, Xue Li, Oswens Siu-Hung Lo, Wai-Lun Law, Lui Ng

**Affiliations:** Department of Surgery, Li Ka Shing Faculty of Medicine, The University of Hong Kong, Hong Kong, China

## Abstract

Change in gene expression is inevitable in cancer development. With more studies demonstrating the contributions of cancer stem cells (CSCs) in colorectal cancer (CRC) development, this study is aimed at investigating whether rectal swab specimen serves as a tool for detection of dysregulation of CSC or stem cell (SC) markers and at evaluating its potential as a new promising screening method for high-risk patients. Expression levels of 15 pluripotency-associated genes were assessed by quantitative PCR in 53 rectal swab specimens referred for endoscopic screening. Dysregulated genes and joint panels based on such genes were examined for their diagnostic potentials for both polyp and CRC. Out of 15 genes, *Oct4*, *CD26*, *c-MYC*, and *CXCR4* showed significantly differential expression among normal, polyp, and CRC patients. A panel of *Oct4* and *CD26* showed an AUC value of 0.80 (*p* = 0.003) in identifying CRC patients from polyp/normal subjects, with sensitivity and specificity of 84.6% and 69.2%. A panel of *c-MYC* and *CXCR4* achieved CRC/polyp identification with an AUC value of 0.79 (*p* = 0.002), with a sensitivity of 82.8% and specificity of 80.0%. The sensitivity for polyp and CRC was 80.0% and 85.7%, respectively. Further analysis showed that higher *c-MYC* and *CXCR4* level was detected in normal subjects who developed polyps after 5-6 years, in comparison with subjects with no lesion developed, and the AUC of the *c-MYC* and *CXCR4* panel increased to 0.88 (*p* < 0.001), with sensitivity and specificity of 84.4% and 92.3%, respectively, when these patients were included in the polyp group. This study suggests that the *Oct4* and *CD26* panel is a promising biomarker for distinguishing CRC from normal and polyp patients, whereas the *c-MYC* and *CXCR4* panel may identify polyp and CRC from normal individuals.

## 1. Introduction

Colorectal cancer (CRC) has become the third most prevalent cancer worldwide in males and second in females, reaching over 1.8 million incidences and 0.8 million deaths in total in 2018 [1]. With an up-growing trend of cases since the 1980s, case number was estimated to reach 2.2 million in 2030 [2]. CRC patients are expected to achieve favourable survival if being diagnosed in an early stage, yet the disease remains asymptomatic in the majority of cases until CRC progress to advanced status. In statistics, only 39% of CRC patients were diagnosed at their less-invasive localized stage, when their five-year survival rate reaches 90% [3]. Such rate was reduced to 71% and, drastically, to 14% for regional and distant stage of cancer. These emphasized the essentials of early detection of colorectal dysplasia, including polyps, adenoma, and CRC, for patients to reduce both chances of developing tumor and severity of cancer if present.

Colonoscopy is considered to be the gold standard tool for the detection of abnormalities in the colon and rectum. Yet, such a procedure requires a significant amount of time per patient, which becomes an obstacle to achieve a large population screening of CRC. In addition, the public may resist colonoscopy due to anxiety, cost, fear of pain during the procedure, feeling uncomfortable during bowel preparation such as fasting and laxative consumption, or just lack of motivation to undergo the procedure [4]. Hence, searching for a noninvasive and cost- and time-effective test is necessary to promote the number of participants of the screening programme for early detection of CRC. With a high specificity and sensitivity test, only individuals diagnosed with lesions will undergo colonoscopy, whereas further checkups are not necessary for the healthy population.

Genetic alterations are inevitable in carcinogenesis, leading to changes in the expression of elements that maintain normal cell functioning, such as oncogenes, tumor suppressor genes, and many gene regulators. Such topics had been extensively studied in CRC, where genetic alterations were detected in the process of transformation of normal cells towards tumors. Some of the markers showed potentials as predictors for chemoresistance of tumor against therapy or as indicators of prognosis of patients [5, 6]. In addition to CRC markers, stem cell (SC) markers and cancer stem cell (CSC) markers may play a vital role in tumorigenesis. Change in expression of such markers were surveilled: *Oct4*, *SOX2*, *CD133*, and *c-MYC* are well-studied SC markers which regulate pluripotency, cell growth, and differentiation [7]; overexpression of *Lgr5*, a WNT signaling pathway regulator, was detected in CRC patients [8–10]; *LRIG1* was suggested to be responsible for stemness of intestinal SC, with ability to prevent epithelial-to-mesenchymal transition in breast epithelial cells [10, 11]; *Msi1* was detected in both nearby Paneth cells where SCs are thought to locate and in stage II CRC tissues [12, 13]; *EpCAM* was associated with the WNT signaling pathway and involved in cell migration and proliferation [7, 9]; CSC cell surface marker *CXCR4* was involved in regulating detachment and migration of cancer cells [14]; considered to be both SC and CSC marker, *ALDH1* is responsible for oxidation of certain aldehydes to carboxylic acid [15]; multiple cluster of differentiation (CD) family members, namely, *CD24*, *CD26*, *CD29*, *CD44*, and *CD166*, were proposed to be crucial for cells to exhibit CSC properties, such as tumor initiation, colony formation, survival, and metastasis, in addition with potential to promote aggressiveness of tumor by promoting cell migration and invasiveness [9, 15–18]. These components drive the stemness of CSCs, potentially leading to the progression of tumor to a later stage.

DNA methylation is currently one of the most investigated mechanisms of epigenetic regulation. By the addition of methyl group (-CH_3_) to cytosine (usually at CpG dinucleotides) of either exon, promoter, or regulatory element of genes, this could result in gene silencing, which plays a vital role in cell development as well as tumorigenesis [19]. Previous studies confirmed that expressions of multiple stem cell markers are under the control of such mechanism during embryo development. For example, promoters of *Oct4* and *Nanog* were observed to be methylated during the differentiation of mouse embryonic stem cells, reducing the pluripotency of the cells, whereas the differentiated cells maintain the hypermethylated state in the promoters of *SOX2*, *c-MYC*, and *Oct4* [20, 21]. Demethylation of lineage-specific genes is also essential for differentiation, allowing their consistent expression for lineage maintenance [19]. Alternatively, it was considered that dysregulation of DNA methylation may contribute to tumor formation. Studies observed the unmethylation of promoter CpG island of *EpCAM* in tumor tissues including CRC, in comparison to the methylated normal samples, yet it was argued that additional factors are required for its overexpression in CRC [22, 23]; although it is not studied in CRC, higher methylation frequency is linked to *ALDH1* suppression in breast cancer, whereas promoter of *CD26* is hypermethylated in melanoma cells [24, 25]. In breast cancer, DNA methylation is also responsible for the dysregulation of *CD44* and *Msi1* [26]. Such findings suggested that changes in expression of pluripotency markers could be driven by such aberrations in epigenetic modification and result in tumor formation.

Rectal swab is a useful tool to study the microenvironment of the rectum. Interestingly, conflicting results were shown in microbial studies: certain strains of bacteria showed different patterns among mucosal biopsy, stool, and swab samples, whereas some presented consistent trends in these samples [27, 28]. Despite the variations, swab samples showed potentials as a method for screening polyps and predicting CRC risk based on microbiome status [27, 29]. In addition, a consistent result was shown in gene expression analysis between tissue biopsy and rectal swab samples [30]. Therefore, we hypothesized that rectal swab could be an effective, noninvasive tool to detect the presence of polyp or CRC. At present, no promising diagnostic panel of genes was suggested for the rectal swab to detect abnormalities in the intestine. Hereby, in this study, we aim to investigate whether dysregulation of stem cell markers, including *Oct4*, *ALDH1A1*, *CD133*, *CD166*, *CD24*, *CD26*, *CD29*, *CD44*, *c-MYC*, *CXCR4*, *EpCAM*, *LGR5*, *LRIG1*, *Msi1*, and *SOX2*, is detected in patients with CRC and to evaluate if rectal swab samples may serve as a tool for gene expression analysis and CRC screening.

## 2. Materials and Methods

### 2.1. Patients and Rectal Swab Specimens

The study consisted of 53 patient subjects (18 normal subjects, 17 subjects with polyp, and 18 CRC subjects) who underwent colonoscopy between 2011 and 2014 in Queen Mary Hospital, Hong Kong. Clinical features of the subjects are described in Table 1. Regarding the CRC subjects, 13 were diagnosed with early-stage tumor (stages I and II) and 5 with late-stage tumor (stages III and IV). No significant difference in gender and age among groups was identified.

Three soft brushes were inserted approximately 2 cm into the rectum of participants, individually using a proctoscope, and gently swabbed the luminal surface for collection of mucosal cells and any mucus or excrete. They were transferred to RNAlater® Solution (Ambion, Austin, TX, USA), by swirling and dipping in the solution and stored at -80°C. Clinicopathological information of the patients was obtained from the clinical management system of the hospital. Follow-up checks of the subjects were performed 5 years postcolonoscopy. The study was approved by the Institutional Review Board, The University of Hong Kong, and informed written consent was obtained from all study participants.

### 2.2. RNA Extraction and Preparation of RNA

Total RNA was extracted using PureLink™ RNA Mini Kit (Ambion) according to the manufacturer's guidelines. RNA samples were eluted in RNase-free water, followed by the measurement of RNA yield and quality using NanoDrop 2000 (Thermo Fisher Scientific, Waltham, MA, USA).

### 2.3. cDNA Synthesis and Quantitative Real-Time Polymerase Chain Reaction

RNA (25 ng) was reversely transcribed to cDNA using PrimeScript RT reagent kit with gDNA eraser (Perfect Real Time) (Takara Bio, Otsu, Japan), to generate a 20 *μ*l genomic DNA-free cDNA according to the manufacturer's instructions. Then, cDNA underwent quantitative PCR amplification with gene-specific oligonucleotide primers using TB Green Premix Ex Taq II (Tli RNaseH Plus) (Takara Bio) following the instructions of the manufacturer. 0.5 *μ*l of gDNA-free cDNA and 0.4 *μ*M of both forward and reverse primers were used per 10 *μ*l reaction mix per well of a 96-well plate. Fifteen genes that are involved in pluripotency of normal cells or CRC were selected, namely, *Oct4*, *ALDH1A1*, *CD133*, *CD166*, *CD24*, *CD26*, *CD29*, *CD44*, *c-MYC*, *CXCR4*, *EpCAM*, *LGR5*, *LRIG1*, *Msi1*, and *SOX2*. The housekeeping gene *GAPDH* was also selected. Quantitative PCR was performed using the ViiA7 Real-Time PCR System (Thermo Fisher Scientific) at 95°C for 30 sec, followed by 40 cycles at 95°C for 5 sec and 60°C for 34 sec. Each assay was done in duplicate, and results were normalized to the expression of *GAPDH* and expressed as −*Δ*Ct (negative delta Ct). Primer sequences used are listed in Supplementary Table 1.

### 2.4. Statistical Analysis

One-way ANOVA test was performed to compare the differences in target mRNA expression among rectal swab specimens of disease-free control subjects, polyp subjects, and CRC patients. To assess the sensitivity and specificity of gene biomarkers and cut-off value for the diagnosis of CRC, receiver operating characteristic (ROC) curves and the area under the ROC curve (AUC) were used. Logistic regression was used to develop a joint mRNA panel to diagnose the presence of CRC. All of these statistical analyses were performed with SigmaPlot 10.0 (Systat Software Inc., San Jose, CA, USA). Statistical significance was set at *p* ≤ 0.05.

## 3. Result

### 3.1. Expression of the 15 Stem Cell-Associated Genes in Normal Subjects and Polyp and CRC Patients

**Figure 1:**
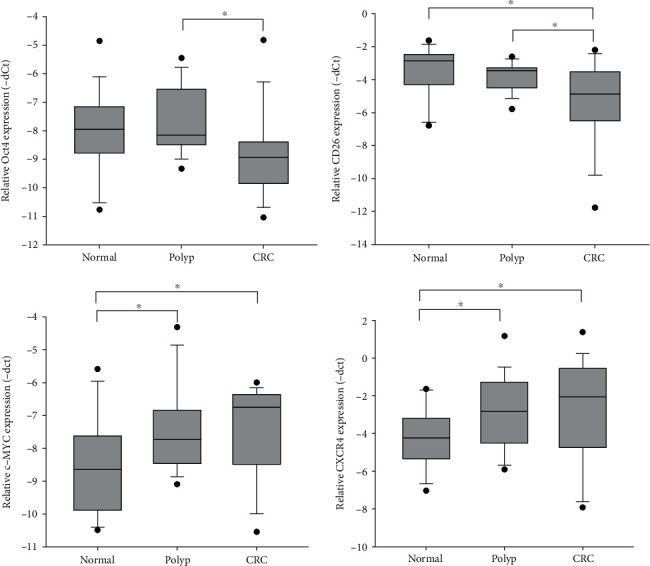
Four pluripotency-associated genes showing significant differential expression pattern among normal, polyp, and CRC patients.

18 normal subjects, 17 polyp patients, and 18 CRC patients participated in the study. After RNA extraction from swab samples and subsequent cDNA synthesis, quantitative PCR was applied to analyze the expression of 15 candidate pluripotency-associated genes including *Oct4*, *ALDH1A1*, *CD133*, *CD166*, *CD24*, *CD26*, *CD29*, *CD44*, *c-MYC*, *CXCR4*, *EpCAM*, *LGR5*, *LRIG1*, *Msi1*, and *SOX2*. One-way ANOVA analyses demonstrated that *Oct4*, *CD26*, *c-MYC*, and *CXCR4* showed significantly differential expression among the three groups (Figure 1). A detailed comparison between normal/polyp, normal/CRC, and polyp/CRC for each gene is shown in Table 2. In particular, both *Oct4* and *CD26* were significantly downregulated in CRC compared to polyp samples. *CD26* also showed reduced expression in CRC compared to the normal group. Alternatively, *c-MYC* and *CXCR4* were upregulated in both polyp and CRC samples, compared to the normal group. Hence, these 4 genes were chosen for downstream investigations.

### 3.2. Potential of the Selected Pluripotency-Associated Genes as a Diagnostic Biomarker

We thus evaluated the AUC, sensitivity, and specificity of these genes in diagnosing CRC using ROC analysis.

**Figure 2:**
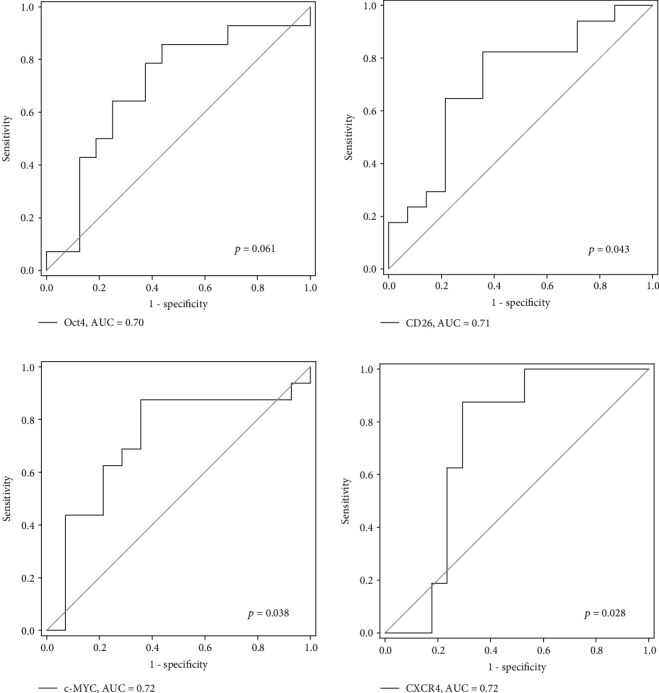
ROC analyses of *Oct4*, *CD26*, *c-MYC*, and *CXCR4* for CRC identification from normal patients.

**Figure 3:**
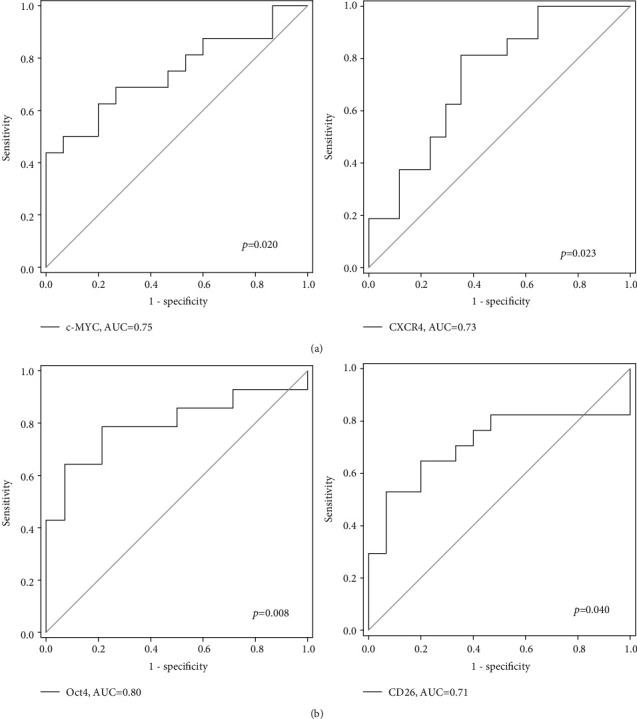
ROC analyses showing that (a) *c-MYC* and *CXCR4* can identify polyp from normal patients and (b) *Oct4* and *CD26* can identify CRC from polyp patients.

We first determined the diagnostic potential of these genes in identifying polyp and CRC patients. *CD26*, *c-MYC*, and *CXCR4* were able to identify patients with CRC from normal patients with AUC values of 0.71, 0.72, and 0.72, respectively (*p* < 0.05, Figure 2). *Oct4* also showed potential to identify CRC patients with an AUC value of 0.70 though the *p* value (0.061) was slightly above the significant threshold (*p* < 0.05). Moreover, as shown in Figure 3(a), *c-MYC* and *CXCR4* were able to identify patients with polyp from normal subjects with AUC values of 0.75 and 0.73, respectively, whereas *Oct4* and *CD26* can identify CRC patients from polyp patients with AUC values 0.80 and 0.71 (Figure 3(b)), respectively (all *p* < 0.05).

**Figure 4:**
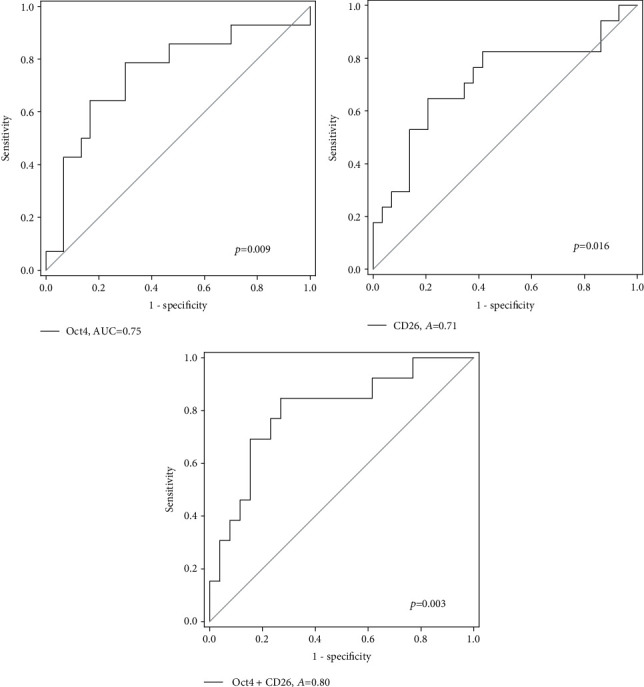
ROC analyses of *Oct4* and *CD26* and their combination for identification of CRC patients from normal and polyp patients.

Furthermore, we evaluated the diagnostic potential of these genes in identifying CRC from normal and polyp subjects (Figure 4). *Oct4* and *CD26* were able to identify CRC patients from polyp/normal subjects with AUC values of 0.75 and 0.71, respectively (*p* < 0.05). A panel of these 2 genes showed an AUC value of 0.80 (*p* = 0.003), with sensitivity and specificity of 84.6% and 69.2%, respectively. The positive predictive value was 61.1% and the negative predictive value was 90.9%. No significant result was shown by such panel in distinguishing normal and polyp subjects (data not shown).

**Figure 5:**
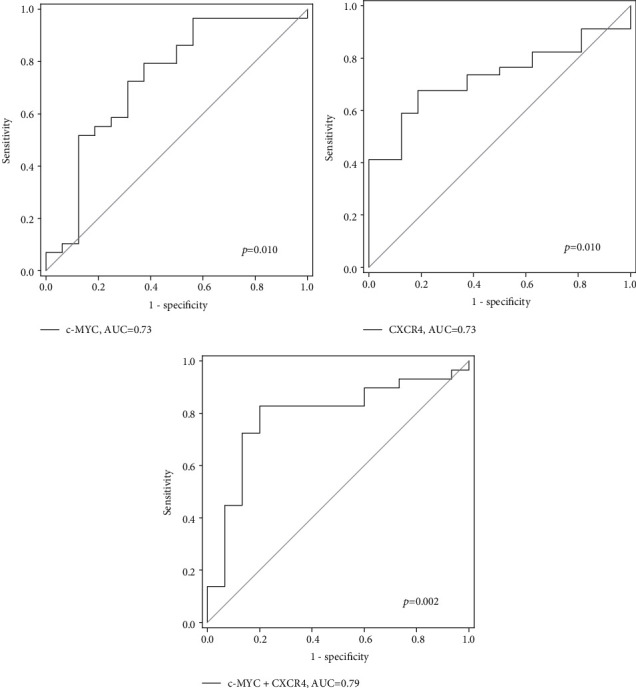
ROC analyses of *c-MYC* and *CXCR4* and their combination for identification of CRC and polyp patients from normal patients.

More importantly, *c-MYC* and *CXCR4* were able to identify polyp/CRC patients with AUC values of 0.73 and 0.73, respectively (*p* < 0.05, Figure 5). A panel of *c-MYC* and *CXCR4* further increased the AUC to 0.79 (*p* = 0.002), with sensitivity and specificity of 82.8% and 80.0%, respectively. The positive predictive value was 89.7% and the negative predictive value was 70.6%. The sensitivity of this panel for polyp and CRC detection was 80.0% and 85.7%, respectively. No significant result was shown by such panel in distinguishing polyp and CRC subjects (data not shown).

Further evaluation of alternative combinations of 2-gene panels, as well as 3-gene and 4-gene diagnostic panels, was performed. Yet, their sensitivity and specificity for polyp and CRC identification did not outperform the two gene panels mentioned above (data not shown). Thus, we confirmed that the panel of *Oct4* and *CD26* was able to identify CRC patients, and the panel of *c-MYC* and *CXCR4* was able to identify polyp/CRC patients with a better performance among different gene sets.

### 3.3. Evaluation of Stage-Associated Change in Panel Value in Early/Late-Stage Patients

**Figure 6:**
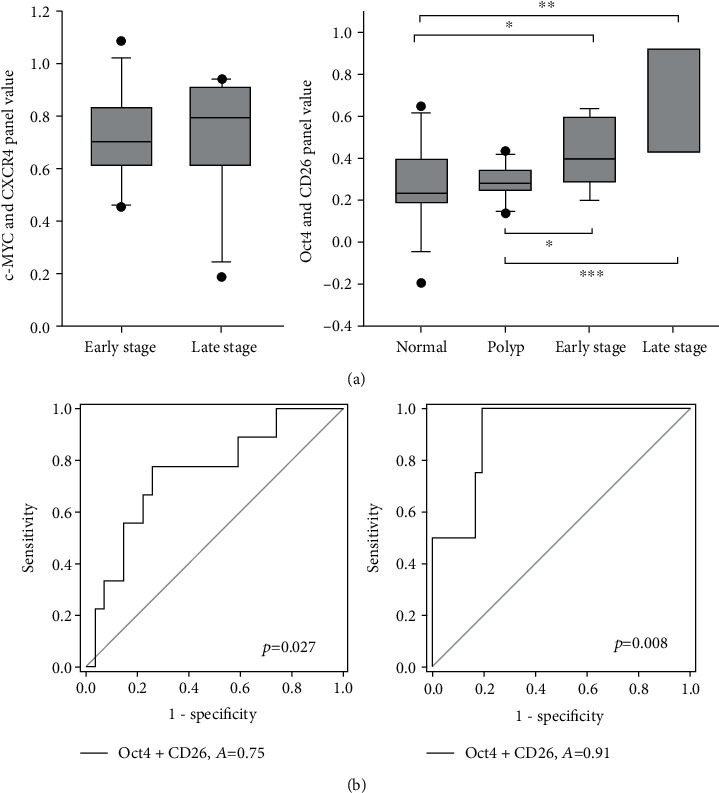
Identification of stage-dependent variation in gene panel values. (a) Comparison of the median value of normal, polyp, and early-stage and late-stage CRC patients in both Oct4 and CD26 panel and c-MYC and CXCR4 panel. (b) ROC analyses of Oct4 and CD26 panel for identification of early-stage CRC patients from normal and polyp patients (left) and late-stage CRC patients from normal, polyp, and early-stage CRC patients (right).

As it is a progressive step of cancer development from polyp to early-stage and eventually to a late-stage tumor, we further compared the panel value of early (*n* = 13, stages I and II) and late-stage (*n* = 5, stages III and IV) CRC patients in both 2-gene panels. A panel value is a composite score calculated by applying the Ct values of gene expressions to the multiple linear regression model. No stage-dependent variation was detected in the *c-MYC* and *CXCR4* panel value, whereas a trend of increasing panel value in late-stage cases was observed in the *Oct4* and *CD26* panel, although the result was not significant (*p* = 0.059, Figure 6(a)). Hence, we compared the values of the *Oct4* and *CD26* panel of the normal, polyp, and early- and late-stage CRC and observed a stepwise increase in the median when CRC develops (0.232, 0.281, 0.393, and 0.648, respectively). Thus, we evaluate if our panel of *Oct4* and *CD26* can identify either early- or late-stage patients from the other subjects. The 2-gene panel was able to distinguish the late-stage CRC patients from the normal, polyp, and early-stage patients with an increased AUC value of 0.91, whereas the identification of early-stage patients from normal and polyp case showed an AUC value of 0.75 (*p* < 0.05, Figure 6(b)).

### 3.4. Predictive Potential of *c-MYC* and *CXCR4* for Polyp Formation in Normal Subjects

**Figure 7:**
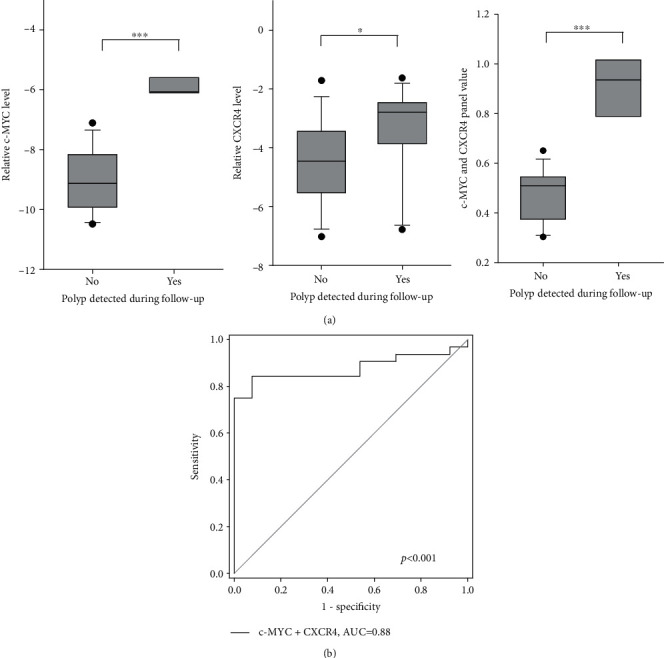
Prognostic significance of *c-MYC* and *CXCR4* and their panel. (a) Comparison of level of *c-MYC* and *CXCR4* and their panel in normal patients with or without polyp detected during follow-up. (b) ROC analyses of *c-MYC* and *CXCR4* panel when patients with polyp detected during follow-up were included in the polyp and CRC groups.

Among the 18 normal subjects who appeared normal during the endoscopic examination, polyp was identified in 3 of the subjects during follow-up colonoscopy 5 years after initial examination. Hence, we compared the expression of *c-MYC* and *CXCR4* between these 3 patients and the other normal subjects. The expression of both *c-MYC* and *CXCR4* was significantly higher in those who developed polyp (Figure 7(a)). Moreover, the value of the 2-gene panel was also significantly higher (0.913 vs. 0.475, *p* < 0.001).

We reperformed the ROC analysis for *c-MYC*, *CXCR4*, and their combination by including the patients who developed polyp during follow-up in the polyp and CRC groups. The *c-MYC* and *CXCR4* panel was able to identify polyp/CRC patients with AUC of 0.88 (*p* < 0.001), with sensitivity and specificity of 84.4% and 92.3%, respectively (Figure 7(b)). The positive predictive value was 96.4% and the negative predictive value was 70.6%. The sensitivity of this panel for polyp and CRC detection was 83.3% and 85.7%, respectively.

## 4. Discussion

Among cancer patients, CRC is the second leading cause of cancer death worldwide [1]. Early diagnosis of CRC or polyp is vital to elevate the survival rate of patients, yet this faces challenges as both polyp and early-stage CRC cases are usually asymptomatic, until the disease progress to a regional or distant stage. The average 5-year survival rate at these stages is low due to invasion of tumor through the intestinal wall and spreading to distant regions; therefore, an effective screening programme will be beneficial to patients. Traditional colonoscopy is considered to be invasive, unpleasant, and time-inefficient. The noninvasive fecal occult blood testing (FOBT) was suggested to achieve only 71.2% and 80.1% sensitivity for proximal and distal CRC, respectively [31]. Even more concerning, a meta-analysis by Rahman et al. measured a 31% pooled sensitivity by FOBT between 2013 and 2018, with individual studies showing sensitivity with a range from 7.4% to 75.0% [32]. Yet, with inconsistent sensitivity, the FOBT may still reduce CRC mortality by 22% [33]. Such data emphasized the importance of developing a higher sensitivity screening tool for effective detection of CRC. Extensive works have been done on understanding tumorigenesis of CRC on a molecular basis, leading to the identification of biomarkers that were used to characterize tumor cells in tissues. Such knowledge was applied to histochemistry staining of biopsy to identify the presence or even severity of tumor in biopsies. This may also be applicable in developing a noninvasive screening tool as studies reported the homogeneity of gene expression in rectal swab samples and tissue biopsy [30, 34]. Hereby, we investigated the possibility of using noninvasive rectal swab specimens to measure CSC biomarker expression for CRC detection.

Colorectal cancer stem cells (CRCSCs) are considered to drive tumor growth without regulation, leading to slow and abnormal growth [35]. Multiple genes were proposed to be valuable in identifying CRCSC in biopsy samples. For example, Piero et al.'s research highlighted the potential of a panel of high *EpCAM*, *CD44*, and *CD166* as a decent CRCSC indicator, whereas other studies suggested *Lgr5* and *ALDH1* having the same value [36–38]. As the tumor was proposed to be developed from CRCSCs and adenocarcinoma from polyp, we may identify individuals with a higher risk of having polyp or CRC if these biomarkers are detected. We selected fifteen genes, which were discussed in other publications regarding their contributions to pluripotency in either SC or CSC, to be investigated. Gene expression analysis of our rectal swab specimens demonstrated that *Oct4*, *CD26*, *c-MYC*, and *CXCR4* presented a different pattern among normal, polyp, and CRC groups. Higher *c-MYC* and *CXCR4* expression was detected in polyp and tumor swab samples compare to normal, whereas expression of *CD26* showed in CRC samples is reduced, in comparison to normal and polyp samples. *Oct4* was downregulated in tumor samples compared to polyps, despite the difference between normal and polyp or CRC being not statistically significant. This suggested the potential of these genes being a biomarker for CRC patient identification. We calculated that the AUC values of *CD26*, *c-MYC*, and *CXCR4* reached 0.71, 0.72, and 0.72, respectively, whereas the panel of *c-MYC* and *CXCR4* promotes AUC to 0.79 with sensitivity and specificity of 82.8% and 80.0%, respectively. The 2-gene panel was also able to predict the normal subjects who would develop polyps, with an improved AUC value of 0.88 and 84.4% sensitivity and 92.3% specificity. Here, we demonstrated that *c-MYC* and *CXCR4* dysregulation can be used to detect the existence of polyp and CRC. Furthermore, we identified the progressive increase in panel value of *Oct4* and *CD26* in normal, polyp, and early-stage and late-stage CRC. Our 2-gene panel of *Oct4* and *CD26* was able to identify late-stage patients with AUC of 0.91 and early-stage with 0.75. Although the sample size for late-stage patients was small, this may provide an insight into distinguishing more severe CRC cases using rectal swab. Expansion of the sample population will be necessary for further studies to evaluate its performance.

As mentioned, *Oct4* is one of the fundamental factors responsible for the regulation of pluripotency, cell growth, and differentiation. *Oct4* was reported to upregulate the expression of *β*-catenin and subsequently interfere with the canonical WNT signalling pathway which plays a vital role in maintaining pluripotency [39, 40]. Previous CRC studies showed that knockdown of *Oct4* would reduce invasiveness (to the liver in a mouse model), survival (*in vitro*), and CSC marker expression, as well as promote apoptosis in colorectal cancer cell lines [40, 41]. However, contradicting results were recently published, suggesting *Oct4* transcript, *Oct4B1*, promotes *Polo-like kinase 1* (*PLK1*) expression which drives EMT regulation when overexpressed [42]. Further research indicated the positive correlation between *Oct4* expression and recurrence (of right-sided colon cancer), metastasis, and TNM stage [7, 43–45]. EMT is known to be one of the key routes for CRC to achieve metastasis. These studies suggested the importance of *Oct4* in the invasiveness of the tumor.

Another Yamanaka factor, c-MYC, is also involved in the WNT signalling pathway as a mediator in CRC, where its upregulation was often detected [46, 47]. It was suggested that it interacts with the promoter of both *Deptor* and *hUTP14a*, increasing their expression and subsequently promoting CRC tumor growth [48, 49]. hUTP14a can also stabilize c-MYC protein, increasing its oncoprotein effects [49]. In addition, suppression of *c-MYC* was suggested to have antiangiogenic effects via enhancing thrombospondin-1 (THBS1) and connective tissue growth factor (CTGF) [50]. *c-MYC* may also be involved in the spreading of CRC, suggested by a higher *c-MYC* expression in liver metastases comparing with primary CRC [51]. Interestingly, it was reported that overexpression of *c-MYC* is associated with better survival of CRC patients and drives the c-MYC-dependent apoptosis [52–54]. Such findings were contrary to the previous concept of stepwise increase of its expression during CRC progression, despite the similar expression in primary and metastatic tumors [55, 56]. A meta-analysis also suggested no correlation between *c-MYC* expression level and prognosis [57]. Nevertheless, it was generally considered that upregulated *c-MYC* can be detected in CRC cases and therefore was selected as a candidate in our study.

CD26, a member of the cluster of differentiation family, can be found in a subpopulation of CRC. An *in vitro* study suggested that downregulation of tumor suppressor tp53, a relatively well-known feature of multiple cancer types, would cause the development of CD26-positive cell subpopulation from CD26-negative parental cancer cells [58]. Studies had indicated the correlation between high *CD26* expression and the potential of metastasis. Distant metastasis was developed in patients with CD26-positive tumor in 8- to 15-month follow-up session, whereas CD26-negative CRC patients showed no metastasis [18]. An *in vitro* study also suggested that increased invasiveness and chemoresistance can be achieved by the CD26-positive patient-derived colorectal stem cells [18]. Further study suggested the positive correlation of CD26 expression with the metastatic ability and poorer tumor differentiation [59]. In line with such findings, it was suggested that higher plasmatic CD26 activity is associated with a poorer survival rate [60].

CD26 was also reported to be indirectly linked to CXCR4 via chemokine SDF-1*α* in immune cell lines, in which CXCR4 is involved in tumorigenesis [61]. CXCR4 was suggested to have a critical role in liver metastasis of CRC cells by driving the transformation of hepatic stellate cells to cancer-associated fibroblasts (CAF) via CXCR4/SDF-1/TGF-*β*1 intercellular signalling [62]. It also assists the differentiation of mesenchymal stem cells to CAF, which may also promote tumor growth and metastatic ability [63]. Together with CXCL12, a ligand for CXCR4, it promotes the expression of *miR-125b* and subsequently leads to EMT and invasion of CRC, as well as forming of a positive feedback loop to promote *CXCR4* expression [64]. Patients with a *CXCR4*-high CRC, according to meta-analyses, are likely to show a poorer prognosis, poorer tumor differentiation, and a higher risk of metastasis [65, 66].

Our result suggested that the *CXCR4* and *c-MYC* panel may identify both polyp and CRC samples, suggesting their involvement in polyp development and potentially in early carcinogenesis. However, limited studies were available to investigate the role of *CXCR4* in tumor initiation. Two researches demonstrated that no significant difference in either RNA or protein CXCR4 expression was identified in polyp tissue samples, in comparison with normal tissues [67, 68]. One *in vivo* study suggested that a subpopulation of CRC, the CD133(+)CXCR4(+) colorectal cancer tumor-initiating cells (Co-TICs), presented a higher tumor formation capacity in a humanized orthotopic mouse model [69, 70]. The involvement of *c-MYC* in tumorigenesis, on the other hand, was investigated in depth. Since 1986, elevated expression of c-MYC protein was detected in polyp tissue using IHC [71]. Further study suggested that the level of c-MYC was correlated to the grade of differentiation of adenomas, as well as their size and malignant potentials [72, 73]. Furthermore, hypomethylation of the exon of *c-MYC* was detected not only in CRC tissue but also partially in polyps, suggesting its deregulation in the dysplasia [74]. High expression of *c-MYC* was also linked to reduced apoptosis in CRC, which is a hallmark of cancer development and progression [75].

We compared trends of *Oct4*, *CD26*, *c-MYC*, and *CXCR4* in rectal swab samples with studies published. *c-MYC* was shown to be upregulated in CRC tissue compared with the adjacent normal specimen, in line with our result [76]. However, regarding the clinical outcomes, contrary suggestions were raised about whether the level of *c-MYC* would affect the prognosis and survival of patients [52, 53, 57]. Uniform results were shown for *CXCR4*: in agreement with our result, elevated *CXCR4* expression was measured in CRC tissues compared with paired normal samples, with the level associated with tumor size, metastasis status, and invasion ability [77, 78]. Alternatively, studies of *Oct4* and *CD26* expression in tissue presented different trends compared with our rectal swab study. A progressive increase of *Oct4* expression was previously shown from normal to polyp tissues and from polyp to CRC tissues [43]. Expression of *Oct4* was also associated with late TNM staging and metastatic ability of tumor, especially towards the liver [43, 44]. In contrast, *Oct4* expression in our CRC swab samples was significantly reduced compared with polyp samples but not normal samples, and there is no significant difference between normal and polyp samples. Our previous study also observed a reduction in *CD26* expression in CRC patient rectal swab, contrary to the study of tissue biopsies which suggested no significant variation between normal and tumor samples [18]. In the literature, serum soluble CD26 reduction was observed in CRC patients compared with normal samples, which was consistent with the swab result [79, 80]. This seemed to be contradicting with the previous findings which suggest higher invasiveness in *CD26*-positive CRC cells [18, 59]. We hypothesize that tumor cells with the CSC marker expression would localize at the central region of primary CRC and daughter cells with low *CD26* expression being released to either lumen of the colorectum or bloodstream after inducing angiogenesis and invasion. Despite a certain degree of contradiction between tissue and rectal swab gene expression, we proposed two new diagnostic panels of two CSC biomarkers to detect polyp and CRC using rectal swab, which presents a decent AUC value (0.80 for CRC only; 0.88 for polyp/CRC).

It is considered that CRC can be developed from polyp; thus, detection of polyp is important to prevent CRC development. Regarding the common noninvasive test, FOBT was reported to show moderate polyp sensitivity of 41.3% for advanced adenomas by Hemoccult SENSA, a high-sensitivity form of guaiac fecal occult blood test (gFOBT) [81]. However, Morikawa's group suggested FOBT showing a lower 10.4% sensitivity for adenoma below 9 mm size and 27.1% for advanced neoplasia, including adenomas above 10 mm size (20.0%) or with severe dysplasia (32.7%) [82]. This was consistent with Imperiale et al.'s study, illustrating the sensitivity of 10.7% for advanced adenoma and 4.8% for minor polyps [83]. Furthermore, the sensitivity of FOBT was strongly accused by Ferlitsch et al., who suggested a stronger correlation between the presence of adenoma and gender over a positive FOBT test [84]. In addition, the ability of the fecal immunochemical test (FIT) to detect adenomas was also investigated. The sensitivity of 30.1% (adenomatous polyp) and 31.1% (neoplastic polyp) was achieved by FIT, according to Chiu et al.'s large-scale study involving over 18,000 participants [85]. Allison et al. supported their findings, showing the 29.5% sensitivity for advanced adenoma detection for FIT [81]. An alternative noninvasive test, the fecal DNA test, also showed a low sensitivity to both advanced adenoma and polyp, accounting for 15.1% and 7.6%, respectively [83]. In short, none of the current noninvasive tests is effective for polyp and adenoma detection. Here, our two-gene panel (*c-MYC* and *CXCR4*) showed a decent 80.0% sensitivity for identifying polyp patients, and the performance further increased to 83.3% when future polyp development was included. This suggested a promising potential of using rectal swab samples for detecting such asymptomatic abnormalities in the colorectum. Progression from adenoma to carcinoma is believed to take an average of at least 10 years [86]. By identifying patients who require polypectomy, this may potentially lower the incidence of CRC development from polyp.

In the mid-2010s, two commercial, noninvasive CRC screening tests were approved by FDA, namely, Cologuard and Epi proColon [87]. Cologuard is a multitarget stool DNA-based test that detects the methylation status of genes *NDRG4* and *BMP3* as well as *KRAS* mutation, in addition to the haemoglobin detection which is similar to FIT. Such test showed an improved CRC and advanced adenoma detection rate, reaching 92.3% and 42.4%, respectively [88]. Yet, researchers suggested that the diseases in other gastrointestinal regions may also affect stool DNA methylation status, which may influence the specificity of the test. Indeed, the specificity of Cologuard for advanced neoplasia was 86.6%, whereas FIT reached 94.9% [87, 88]. Alternatively, Epi proColon is a plasma-based DNA test that analyzes the methylation status of gene *SEPT9* [87]. Comparing with the other tests, the sensitivity of such blood test was relatively poor, with the value of 35% to 77% achieved, depending on the stages of CRC [89]. Although a recent meta-analysis suggested the second-generation Epi proColon test 2.0 showed better sensitivity of 74% for CRC, such a result did not outperform the available tests [90]. Besides, Epi proColon showed a weak sensitivity of 11% for advanced adenomas, suggesting that it is unable to identify precancerous lesions.

In addition to the commercially available tests, increased studies of the development of methylation-based biomarker panels have been done since the 2010s. A study in 2013, involving 87 samples, established a panel of *AGTR1*, *WNT2*, and *SLIT2* in stool DNA in which their methylation status was detected by pyrosequencing assay [91]. In this 87-sample study, the panel achieved 78% sensitivity and 89% specificity for CRC identification. A more recent stool DNA study in 2020 proposed a *mSept9* and *mSDC2* panel detected using qPCR assay, which achieved 66.7% and 92.3% sensitivity for advanced adenoma and CRC in a 106-sample study [92]. In Fadda et al.'s study, by analyzing the CpG islands associated with *GRIA4*, *SLC8A1*, and *SYN3* genes, methylation of at least one marker was observed in 87.5% stool DNA samples from CRC patients [93]. Note that the stool samples were taken during operation, in which the condition of stool could be affected by bowel preparation. Further study is also required to evaluate both the specificity of the panel and the performance in polyp/advanced adenoma identification.

Another source of DNA methylation biomarkers for CRC diagnosis is the blood samples, which had been studied since 2002, when *p16* promoter hypermethylation was detected in serum from CRC patients [94]. Methylation of *Sept9*, which is used in Epi proColon, was first studied in 2007 [95]. Since then, panels of methylation biomarkers have been developed. Two of the best-performing panels were as follows: (1) panel of *APC*, *MGMT*, *RASSF2A*, and *Wif-1*, which can identify CRC cases with 86.5% sensitivity and 92.1% specificity, using methylation-specific PCR [96]; it can also distinguish adenoma patients with 74.6% sensitivity; (2) panel of methylated *C9orf50*, *KCNQ5*, and *CLIP4*, to be detected in plasma using methylation-specific droplet digital PCR, which achieves 85% sensitivity and 99% specificity for CRC [97]. Comparing with these blood-based and stool-based gene panels, not only a competitive sensitivity was achieved by our method but we also evaluated the predictive value of the gene panel, which was lacking from other studies.

When comparing the performance of our proposed rectal swab test and commonly used noninvasive tests (e.g., FIT), indeed, this pilot study did not present an improved performance in CRC detection or data of reduction in mortality. However, rectal swab remains valuable as a potential screening test: a previous small-scale survey has suggested that rectal swab could be more preferable than stool test by the participants and may promote collection rate [98]. Such swab specimens are self-collectable or can be assisted by healthcare staffs if patients face difficulty [99]. In fact, in the study of gastroenteritis, the rectal swab could be more favourable in comparison with the stool test, due to the easier transport and handling [100]. Furthermore, CRC is caused by a cascade of molecular genetic changes in the tissues. Therefore, change of gene expression can be the first marker of CRC, before the presence of occult blood in stool or immune response in the blood. Therefore, detection of such change may allow earlier diagnosis. Here, a rectal swab can directly collect materials from the rectum for gene expression analysis. Such a strategy is not applicable in many other cancer types when cancerous tissues are unreachable noninvasively. The direct collection of samples from the colorectum may also promote the specificity of gene expression to the condition of the organ, rather than the measurement of “secondary” samples such as stool and blood, which may be influenced by factors irrelevant to CRC.

In past publications, the majority of rectal swabs were used in microbial studies. Sequencing (pyrosequencing, amplicon sequencing, ion torrent, etc.) had been extensively used to analyze the presence of different species of bacteria in the intestinal lumen to investigate the difference in composition of them between healthy controls and patients with either colorectal cancer or other diseases [27, 29, 101, 102]. Also, such a technology was used to compare their presence in stool and rectal swab [99, 103]. Alternatively, qPCR was used in other studies to quantify the bacterial 16S rRNA after DNA extraction, to compare biopsy samples and swab samples, or the effects of drug treatments that are aimed at altering the content of the microbiome [28, 104, 105]. Other methods to identify components of microbiome include studying the terminal restriction fragment length polymorphism (T-RFLP) after DNA extraction [28] and inoculation of rectal swab specimens on culture media for growth, followed by a biochemical test, e.g., carbohydrate fermentation and pigment production vancomycin screening test [106]. In terms of nonmicrobial studies, very limited publications were reported to use rectal swab as a tool for gene expression study. It has been reported that qPCR was used to measure the expression of biomarkers by rectal mucosa cells collected from rectal swabs [30]. ELISA was used in another pilot study to quantify the level of eosinophil-derived neurotoxin in patients with allergic proctitis [107]. By comparing different methods used by other researchers, qPCR was considered to be the most appropriate method to quantify the expression of the SC markers of interests, instead of identifying the mutations using the sequencing method or checking for the existence of gene expression using ELISA.

One of the key limitations of our study was the relatively small number of subjects involved in this study. This limited our study to be unable to include additional factors into the study, such as the previous history of gastrointestinal pathology, history of smoking, and alcohol consumption. More importantly, only 3 participants were initially categorised in the normal group and developed polyps within 5 years. Increased population size is necessary to validate the identification of increased *c-MYC* and *CXCR4* expression. With a larger sample size, the *p* value of the panel for distinguishing early-stage cases could also be improved. Yet, our finding served as a pilot study to explore the possibility of using rectal swab to diagnose individuals with polyps or CRC and emphasize the resources available in the microenvironment of the bowel. Our screening of fifteen CSC or SC biomarkers suggested that cells with a certain degree of pluripotency were present in the distant region of the colorectum from the tumor. This brings consideration of the importance of the microenvironment not only in terms of bacteria but also the presence of shedding cells or components. Extensive works are needed to explore potential biomarkers, both genetic and microbial, that can be measured in the lumen of the rectum, before a complete panel to be established for use in the next-generation screening programme.

## 5. Conclusion

To conclude, our study illustrated the potential of using rectal swab to measure the gene expression of multiple cancer-related stem cell markers. Using this method, we detected the upregulation of *c-MYC* and *CXCR4* in both tumor and polyp, as well as downregulation of *CD26* and *Oct4* in tumor when comparing with normal plus polyp and polyp, respectively. This data allows us to design different panels and endue rectal swabs with a new role as a noninvasive screening test for the detection of lesion or CRC. Such an easy-to-achieve method allows the potential of popularization of the screening programme, which may significantly elevate the number of participants and, hopefully, bring us a decline of incidences of CRC. With more CRC and polyp patients identified and treated at an earlier stage, this will minimize the damage done by CRC to individuals and also their families, promoting their quality of life.

## Figures and Tables

**Table 1:** Clinical characteristics of normal, polyp, and CRC patients.

*Normal subjects(n* = 18)		
Gender	Female	7
	Male	11
Age (mean = 64.0)	<65	10
	≥65	8
*Polyp subjects(n* = 17)		

Gender	Female	6
	Male	11
Age (mean = 60.6)	<65	11
	≥65	6
*CRC subjects(n* = 18)		

Gender	Female	6
	Male	12
Age (mean = 69.8)	<65	7
	≥65	11
Location	Proximal colon	5
	Distal colon	7
	Rectum	6
Stage (AJCC)	I	5
	II	8
	III	4
	IV	1
Tumor differentiation	Well	0
	Moderate	16
	Poor	2

No significant difference in gender and age among groups (*p* > 0.05). Proximal colon = caecum, ascending colon and transverse colon; distal colon = descending colon and sigmoid.

**Table 2:** Detailed comparison of *Oct4*, *CD26*, *c-MYC*, and *CXCR4* levels in normal, polyp, and CRC patients.

	Level in normal/polyp/CRC (-delta Ct)	Normal vs. polyp	Normal vs. CRC	Polyp vs. CRC
*Oct4*	-8.056/-7.731/-8.905	NS	NS	*p* = 0.027
*CD26*	-2.872/-3.454/-4.876	NS	*p* = 0.045	*p* = 0.041
*c-MYC*	-8.616/-7.370/-7.421	*p* = 0.019	*p* = 0.032	NS
*CXCR4*	-4.296/-2.792/-2.633	*p* = 0.019	*p* = 0.046	NS

NS: not significant.

## Data Availability

All data generated or analyzed during this study are included in this published article.

## Supplementary Materials

Supplementary Materials Supplementary Table 1: primer sequences of each gene.
