# Supplementary material for: A Pilot Study Investigating the Expression Levels of Pluripotency-Associated Genes in Rectal Swab Samples for Colorectal Polyp and Cancer Diagnosis and Prognosis
Source: Stem Cells Int. 2021 Jul 22;2021:4139528. doi: 10.1155/2021/4139528 (PMC8324395; doi:10.1155/2021/4139528)
Supplement: Supplementary Materials — Supplementary Table 1: primer sequences of each gene. [file 4139528.f1.docx]

**Supplementary table 1: Primer sequences of each gene**

| Oct4 | Forward:  Reverse: | CTTGAATCCCGAATGGAAAGGG  GTGTATATCCCAGGGTGATCCTC |
| --- | --- | --- |
| ALDH1A1 | Forward:  Reverse: | CGGGAAAAGCAATCTGAAGAGGG  GATGCGGCTATACAACACTGGC |
| CD133 | Forward:  Reverse: | CACTACCAAGGACAAGGCGTTC  CAACGCCTCTTTGGTCTCCTTG |
| CD166 | Forward:  Reverse: | TCCAGAACACGATGAGGCAGAC  GTAGACGACACCAGCAACAAGG |
| CD24 | Forward:  Reverse: | CACGCAGATTTATTCCAGTGAAAC  GACCACGAAGAGACTGGCTGTT |
| CD26 | Forward:  Reverse: | AAAGGCACCTGGGAAGTCATCG  CAGCTCACAACTGAGGCATGTC |
| CD29 | Forward:  Reverse: | GGATTCTCCAGAAGGTGGTTTCG  TGCCACCAAGTTTCCCATCTCC |
| CD44 | Forward:  Reverse: | CCAGAAGGAACAGTGGTTTGGC  ACTGTCCTCTGGGCTTGGTGTT |
| c-MYC | Forward:  Reverse: | CCTGGTGCTCCATGAGGAGAC  CAGACTCTGACCTTTTGCCAGG |
| CXCR4 | Forward:  Reverse: | CTCCTCTTTGTCATCACGCTTCC  GGATGAGGACACTGCTGTAGAG |
| EpCAM | Forward:  Reverse: | GCCAGTGTACTTCAGTTGGTGC  CCCTTCAGGTTTTGCTCTTCTCC |
| LGR5 | Forward:  Reverse: | CCTGCTTGACTTTGAGGAAGACC  CCAGCCATCAAGCAGGTGTTCA |
| LRIG1 | Forward:  Reverse: | GTGTCATCACCAACCACTTTGGC  GCAATCTGAGGGTTTGGGTGAC |
| Msi1 | Forward:  Reverse: | GCTCAGCCAAAGGAGGTGATGT  GCGTAGGTTGTGGCTTGGAAAC |
| SOX2 | Forward:  Reverse: | GCTACAGCATGATGCAGGACCA  TCTGCGAGCTGGTCATGGAGTT |
| GAPDH | Forward:  Reverse: | GTCTCCTCTGACTTCAACAGCG  ACCACCCTGTTGCTGTAGCCAA |
